# A comprehensive protocol for ventilator weaning and extubation: a prospective observational study

**DOI:** 10.1186/s40560-019-0402-4

**Published:** 2019-11-06

**Authors:** Kenichi Nitta, Kazufumi Okamoto, Hiroshi Imamura, Katsunori Mochizuki, Hiroshi Takayama, Hiroshi Kamijo, Mayumi Okada, Kanako Takeshige, Yuichiro Kashima, Takahisa Satou

**Affiliations:** Department of Emergency and Critical Care Medicine, Shinshu University School of Medicine, 3-1-1 Asahi, Matsumoto, Nagano 390-8621 Japan

**Keywords:** Comprehensive protocol for ventilator weaning and extubation, Hospital mortality, Post-extubation respiratory failure, Reintubation

## Abstract

**Background:**

Ventilator weaning protocols have been shown to reduce the duration of mechanical ventilation (MV), intensive care unit length of stay, and resource use. However, weaning protocols have not significantly affected mortality or reintubation rates. The extubation process is a critical component of respiratory care in patients who receive MV. Post-extubation respiratory failure (PERF) is a common event associated with significant morbidity and mortality. We hypothesized that a comprehensive protocol for ventilator weaning and extubation would be effective for preventing PERF and reintubation and reducing mortality in critically ill patients.

**Methods:**

A ventilator weaning and extubation protocol was developed. The protocol consisted of checklists across four evaluations: spontaneous breathing trial, extubation, prophylactic non-invasive positive pressure ventilation (NPPV), and evaluation after extubation. Observational data were collected after implementing the protocol in patients admitted to the Advanced Emergency and Critical Care Center of Shinshu University Hospital. Not only outcomes of patients but also influences of each component of the protocol on the clinical decision-making process were investigated. Further, a comparison between PERF and non-PERF patients was performed.

**Results:**

A total of 464 consecutive patients received MV for more than 48 h, and 248 (77 women; mean age, 65 ± 17 years) were deemed eligible. The overall PERF and reintubation rates were 9.7% and 5.2%, respectively. Overall, 54.1% of patients with PERF received reintubation. Hospital stay and mortality were not significantly different between PERF and non-PERF patients (*p* = 0.16 and 0.057, respectively). As a result, the 28-day and hospital mortality were 1.2% and 6.9%, respectively.

**Conclusions:**

We found that the rates of PERF, reintubation, and hospital mortality were lower than those in previous reports even with nearly the same degree of severity at extubation. The comprehensive protocol for ventilator weaning and extubation may prevent PERF and reintubation and reduce mortality in critically ill patients.

## Background

Weaning protocols have become popularized since the publication of guidelines by the task force on ventilator discontinuation in 2001 [1]. Several studies have reported that weaning protocols reduced the total duration of ventilation, weaning duration, and intensive care unit (ICU) length of stay without impacting mortality or adverse events [2–4].

Meanwhile, the extubation process is a critical component of respiratory care in patients who receive mechanical ventilation (MV). Post-extubation respiratory failure (PERF) is a common event associated with significant morbidity and mortality [5]. It can be caused by upper airway obstruction or the inability to protect the airway and clear secretions in addition to causes of ventilator-withdrawal failure [6]. Thus, the decision to extubate requires further assessment of the patient’s ability to avert PERF. Many studies that have assessed the need for artificial airway have been reported [4, 7–9]. However, there is no definite guideline for the extubation process.

Insufficient evidence is available regarding a systematic approach for ventilator weaning and extubation. Therefore, we developed a comprehensive protocol for ventilator weaning and extubation based on the screening of meaningful physiologic and clinical variables followed by a spontaneous breathing trial (SBT). Furthermore, prophylactic use of non-invasive positive pressure ventilation (NPPV) was included in our protocol. We hypothesized that the comprehensive protocol would be effective for preventing PERF and reintubation and reducing mortality of critically ill patients.

## Methods

This prospective observational cohort study included all patients who received MV under tracheal intubation in the Advanced Emergency and Critical Care Center of Shinshu University Hospital. This study was approved by the Ethics Review Board of Shinshu University School of Medicine (Approval Number: 2652). The requirement for informed patient consent was waived since the protocol was deemed critical for improving patient care.

### Patient selection

All patients who required MV under tracheal intubation for 48 h or more between April 2007 and March 2013 at the study center were eligible. During this period, for all consecutive adults, we prospectively implemented a comprehensive protocol for ventilator weaning and extubation. Patients were excluded from the study if they were below 18 years of age, died under MV, received tracheostomy, had self-extubation before or after fulfilling the conditions for SBT, were transferred to our center under MV, or were under a do-not-resuscitate status.

### Comprehensive protocol for ventilator weaning and extubation

**Fig. 1:**
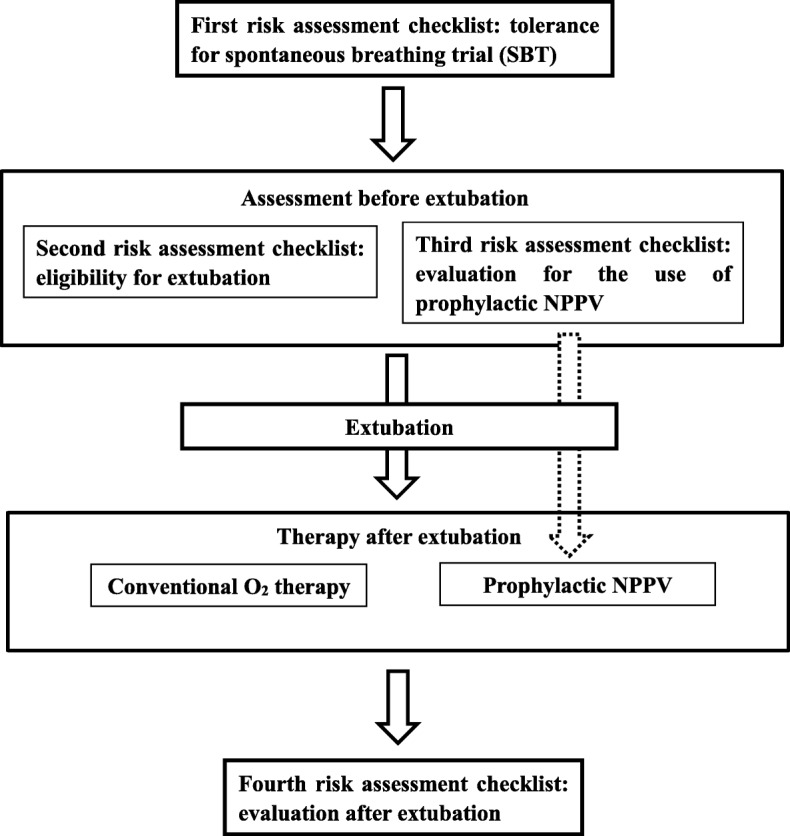
Protocol flow chart. SBT, spontaneous breathing trial; PERF, post-extubation respiratory failure; NPPV, non-invasive positive pressure ventilation

**Fig. 2:**
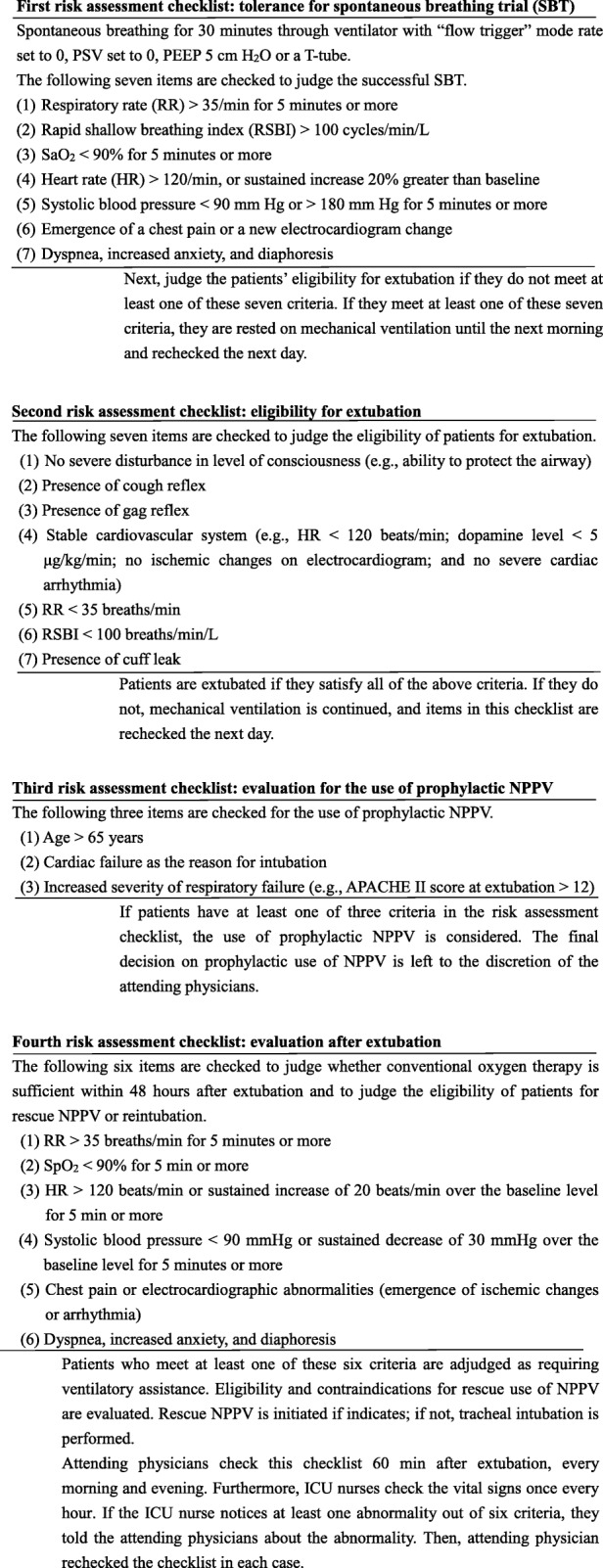
Details of a comprehensive protocol for ventilator weaning and extubation. SBT, spontaneous breathing trial; PSV, pressure support ventilation; PEEP, positive end-expiratory pressure; RR, respiratory rate; RSBI, rapid shallow breathing index; SaO_2_, oxygen saturation; HR, heart rate; PERF, post-extubation respiratory failure; APACHE II, Acute Physiology and Chronic Health Evaluation II; NPPV, non-invasive positive pressure ventilation; SpO_2,_ arterial oxygen saturation

We developed a protocol for ventilator weaning and extubation [1, 10, 11]. This protocol consists of four risk assessment checklists: (1) tolerance of SBT, (2) eligibility for extubation, (3) evaluation for the use of prophylactic NPPV, and (4) evaluation after extubation (Figs. 1 and 2). If the first risk assessment checklist was passed, both second and third checklists were assessed simultaneously. Patients were extubated if they met all seven criteria of the eligibility for extubation (second risk assessment checklist); if not, MV was continued, and items in this checklist were rechecked the next day. If a patient has at least one of the three criteria in the third risk assessment checklist, the use of prophylactic NPPV is considered. The final decision on the use of prophylactic NPPV is left to the discretion of the attending physicians. The evaluation after extubation (fourth risk assessment checklist) involved evaluation within 48 h after extubation. Attending physicians checked this checklist 60 min after extubation, every morning and evening. Furthermore, if the ICU nurses noticed at least one abnormality out of six criteria during the once every hour physical assessment, they told the attending physicians about the abnormality. Then, the attending physician rechecked the checklist in each case. Patients who met at least one of the six criteria of this risk assessment checklist were adjudged as PERF and were administered rescue NPPV or reintubation. Rescue NPPV was applied following the protocol proposed by Kikuchi et al. [12]. The protocol for NPPV comprised of six checklists: (1) the need for ventilatory assistance, (2) the eligibility for NPPV, (3) the effectiveness evaluation at 30–120 min after the start of NPPV, (4) the effectiveness evaluation at 12–24 h after the start of NPPV, (5) the eligibility for weaning, and (6) the evaluation at 30–120 min after the discontinuation of NPPV [12]. We used the first four of the checklists in Kikuchi et al.’s protocol, leaving out the fifth and sixth checklists. For patients who did not fulfill each checklist, reintubation was performed. Patients who did not meet any of the six evaluation criteria for the fourth risk assessment checklist were continued on conventional oxygen therapy. The protocol was executed mainly by residents under the tutoring of intensivists.

### Data collection and outcome variables

In all patients, the following parameters were recorded before extubation: heart rate, mean arterial pressure, respiratory rate, Glasgow Coma Scale score under tracheal intubation (GCS_*T*_ score), arterial blood gas (ABG) analysis, Acute Physiology and Chronic Health Evaluation (APACHE) II score, sequential organ failure assessment score under tracheal intubation (SOFA_*T*_ score), and rapid shallow breathing index (RSBI). Outcome variables included PERF and reintubation rates, lengths of critical care center (CCC) and hospital stay, and 28-day, 60-day, and hospital mortality. While RSBI was defined as the ratio of respiratory rate to tidal volume, GCS_*T*_, and SOFA_*T*_ scores were defined based on the description by Coplin et al. [13]; the verbal component of the GCS was arbitrarily designated as one for patients under intubation. Acute respiratory distress syndrome (ARDS) was defined by PaO_2_/FiO_2_ < 200 regardless of positive end-expiration pressure (PEEP) level, on the basis of a previous definition of ARDS [14]. PERF was defined in the following events: (1) when reintubation was performed within 48 h after extubation, (2) when prophylactic NPPV was required for more than 48 h, and (3) when a rescue NPPV was performed under conventional oxygen therapy within 48 h after extubation.

### Statistical analysis

All patients were analyzed on an intention-to-treat basis. For continuous variables, mean ± standard deviation (SD) or median and 25% and 75% percentile values were calculated. Comparison between the two groups was performed using the Mann–Whitney *U* test. All statistical analyses were performed with EZR (Saitama Medical Center, Jichi Medical University), which is a graphical user interface for R (The R Foundation for Statistical Computing, version 2.13.0) [15]. EZR is a modified version of the R commander (version 1.6–3) that includes statistical functions that are frequently used in biostatistics.

## Results

**Fig. 3:**
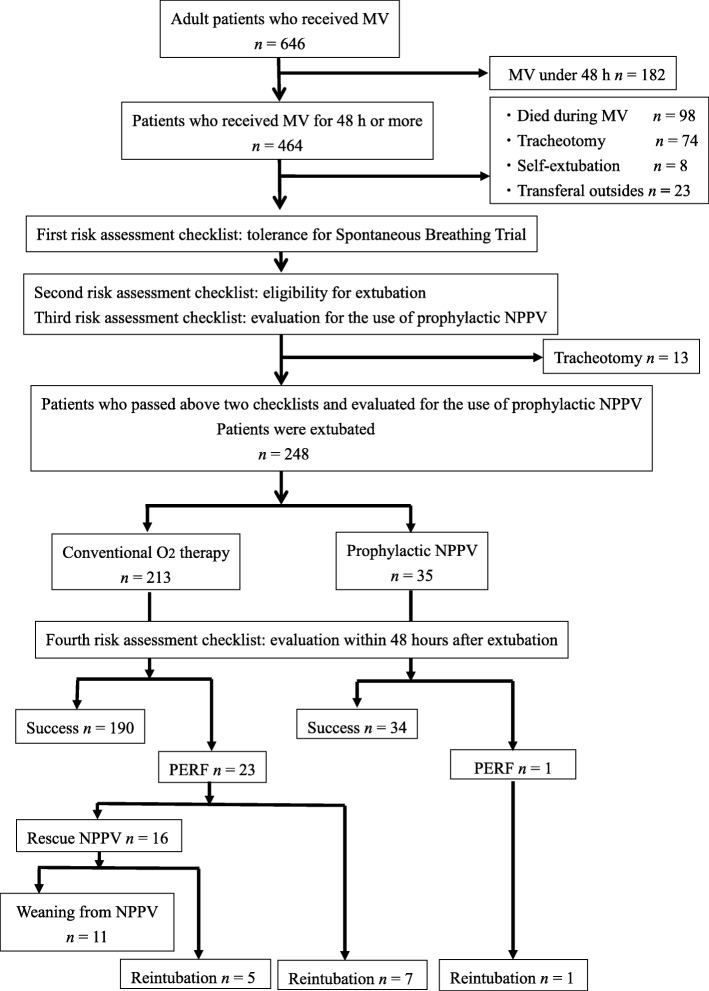
Flow chart of the study patients. MV, mechanical ventilation; PERF, post-extubation respiratory failure; NPPV, non-invasive positive pressure ventilation

Of 464 consecutive patients who received MV for more than 48 h, 216 were excluded for the following reasons: death during MV (*n* = 98), tracheotomy (*n* = 87), self-extubation (*n* = 8), and transfer from the center to the general ward or another hospital under MV (*n* = 23) (Fig. 3). The remaining 248 patients were deemed eligible for this study. Table 1 presents the baseline characteristics of the study population. The median patient age was 65 years (mean age, 65 ± 17 years). Reasons for MV included ARDS (*n* = 159; 64.1%), congestive heart failure (CHF) (*n* = 57; 23.0%), and post-cardiac arrest syndrome (*n* = 30; 12.1%). All 248 patients were extubated after a 30-min SBT and application of the extubation protocol. Of the 248 patients, 213 patients received conventional oxygen therapy, and the remaining 35 patients received prophylactic NPPV.

**Table 1:** Patients’ characteristics

	All patients	PERF	Non-PERF	*p* values
	(*n* = 248)	(*n* = 24)	(*n* = 224)	
Age (years)	65 ± 17	64 ± 18	65 ± 17	0.8
Women, *n* (%)	77 (31)	7 (29)	70 (31)	1.0
APACHE II score just before extubation	13 (10–16)	12.5 (11–16)	13 (10–16)	0.8
SOFA_*T*_ score just before extubation	5 (4–6)	5 (4–6.3)	5 (4–6)	0.5
RSBI (breaths/min/L)	44 ± 21	45 ± 18	44 ± 21	0.9
Duration of mechanical ventilation (days)	7 (5–11)	7.5 (5–13.3)	7 (5–11)	0.6
Reason for mechanical ventilation, *n* (%)				0.2
ARDS	159 (64.1)	14 (58)	145 (65)	
Congestive heart failure	57 (23.0)	5 (21)	52 (23)	
Post-cardiac arrest syndrome	30 (12.1)	4 (16)	26 (12)	
COPD exacerbation	2 (0.8)	1 (4)	1 (0.4)	
Comorbid diseases, *n* (%)				0.4
COPD	17 (7)	5 (21)	12 (5)	
Coronary artery disease	35 (14)	5 (21)	30 (13)	
Chronic heart failure	25 (10)	4 (17)	21 (9)	
Chronic renal failure	14 (6)	1 (4)	13 (6)	
Diabetes mellitus	44 (18)	8 (33)	36 (16)	
Hypertension	85 (34)	9 (38)	76 (34)	
Vital signs just before extubation				
Heart rate (beats/min)	88 ± 19	88 ± 19	88 ± 18	0.9
MAP (mmHg)	90 ± 15	89 ± 16	90 ± 15	0.6
RR (breaths/min)	20 ± 7	20 ± 6	20 ± 7	0.9
ABG values just before extubation				
pH	7.45 ± 0.04	7.45 ± 0.04	7.45 ± 0.04	0.5
PaCO_2_ (mmHg)	38 ± 6	39 ± 5	38 ± 6	0.5
PaO_2_/FiO_2_	301 ± 83	282 ± 88	303 ± 83	0.3

Data are presented as mean ± standard deviation, median and interquartile range, or number (percentage). *PERF* post-extubation respiratory failure, *APACHE II* Acute Physiology and Chronic Health Evaluation II, *SOFA*_*T*_ *score* sequential organ failure assessment score under tracheal intubation, *RSBI* rapid shallow breathing index, *ARDS* acute respiratory distress syndrome, *COPD* chronic obstructive pulmonary disease, *MAP* mean arterial pressure, *RR* respiratory rate, *ABG* arterial blood gas, *PaCO2* partial pressure of carbon dioxide, *PaO*_*2*_ partial pressure of arterial oxygen, *FiO*_*2*_ fraction of inspired oxygen

Overall, 224 patients were successfully extubated, while 24 patients had PERF. There were 23 cases of PERF among the conventional oxygen therapy patients and one of 35 patients who underwent prophylactic NPPV. Of the 24 PERF patients, 13 eventually required reintubation. Of the 23 PERF patients receiving conventional oxygen therapy, 16 received rescue NPPV while 11 patients avoided reintubation. Protocol violation of the tolerance of SBT and eligibility for extubation risk assessments was observed in 20 (8%) patients. Nevertheless, these patients were included in the analyses on an intention-to-treat basis. There was no instance of violation of the evaluation after extubation risk assessment.

The overall PERF and reintubation rates were 9.7% (24/248) and 5.2% (13/248), respectively. The 28-day, 60-day, and hospital mortality rates were 1.2%, 4.4%, and 6.9%, respectively (Table 2).

**Table 2:** Outcome variables

	All patients	PERF	Non-PERF	*p* values
	(*n* = 248)	(*n* = 24)	(*n* = 224)	
Reintubation, *n* (%)	13 (5.2)	13 (54.1)	–	–
CCC stay (days)	20 (12–33)	25 (16.8–47)	19.5 (12–32)	0.058
Hospital stay (days)	33 (21–52)	44 (25.8–57.8)	33 (20–51.3)	0.16
28-d mortality	3 (1.2)	1 (4.1)	2 (0.9)	0.3
60-d mortality	11 (4.4)	1 (4.1)	10 (4.5)	1.0
Hospital mortality	17 (6.9)	4 (16.7)	13 (6.3)	0.057

Data are presented as median and interquartile range or number (percentage). *PERF* post-extubation respiratory failure, *CCC* critical care center, *28-d mortality* 28-day mortality after admission, *60-d mortality* 60-day mortality after admission

### Comparison of patients with PERF (*n* = 24) and non-PERF (*n* = 224)

There was no significant difference in age and sex between the PERF and non-PERF groups (Table 1). There were no significant differences in vital signs and ABG values just before extubation between the two groups. Regarding outcomes, hospital mortality tended to be higher (16.7% vs 6.3%, *p* = 0.057) and the length of CCC stay tended to be longer in PERF patients than in non-PERF patients (Table 2). But there was no difference in 28-day, 60-day mortality, and length of hospital stay between the groups. The reintubation rate was 54.1% in patients with PERF. Reintubated patients had a longer CCC stay than did patients without reintubation after PERF. But there was no significant difference in the length of hospital stay and hospital mortality between patients with and without reintubation. Ten patients with PERF underwent a tracheotomy after reintubation. An additional table file shows this in more detail (see Additional file 1).

### Subgroup analysis: characteristics and outcomes of prophylactic NPPV

Among 248 patients, 35 received prophylactic NPPV. The most common reason for undergoing prophylactic NPPV, at 54% (19/35), was “cardiac failure as the reason for intubation”. Twenty-three patients fulfilled “age > 65 years” and 21 patients fulfilled “APACHE II score at extubation > 12.” Compared to conventional oxygen therapy, prophylactic NPPV was more frequently applied to patients with CHF (*p* < 0.001) and less frequently to patients with ARDS (*p* = 0.001). An additional table file shows this in more detail (see Additional file 2). Compared to conventional oxygen therapy, prophylactic NPPV exhibited significantly higher SOFA_*T*_ scores, RSBI, respiratory rate, PaCO_2_ (*p* = 0.009, 0.003, 0.02, and 0.02, respectively), and significantly lower heart rate and PaO_2_/FiO_2_ ratio (*p* = 0.02 and *p* < 0.001, respectively) just before extubation (Additional file 2). Outcomes did not differ significantly between prophylactic NPPV and conventional oxygen therapy. An additional table file shows this in more detail (see Additional file 3).

## Discussion

This study showed that PERF and reintubation occurred infrequently (9.7% and 5.2%, respectively) and hospital mortality was low (6.9%) by the use of a comprehensive protocol for ventilator weaning and extubation.

Studies have shown that PERF occurs in approximately 15% of cases and is associated with a high mortality rate of 25–50% [10, 16]. Reintubation has a reported association with increased mortality [10, 11, 17, 18] and the extubation failure rate is dependent on the type of patient [19]. PERF occurs in 5–8% of critically ill surgical patients (trauma, cardiothoracic surgery, and general surgery), whereas 12–29% of pediatric, medical, multidisciplinary, and neurologic ICU patients [1, 4, 10, 16, 17, 19, 20]. The PERF rate in our CCC which was categorized as multidisciplinary ICU (Table 1) was less than those of previous studies. Hernández et al. [21] reported that the PERF rate and reintubation rate was 14.4% and 12.2%, respectively, in a control group with a low APACHE II score of 7 at the time of extubation in a study investigating the effect of nasal high flow therapy. Ferrer et al. [11] reported a PERF rate of 33% in a control group with an APACHE II score at extubation of 13 ± 3 in a study investigating the effect of prophylactic NPPV. The PERF and reintubation rates in our study were lower than previous reports [21, 22] even though the APACHE II score just before extubation was 13. In the same study by Ferrer et al. [11], hospital mortality in patients receiving conventional oxygen therapy was 23%. In our study, hospital mortality was lower than that in the aforementioned report even with nearly the same degrees of severity at extubation.

### Impact of the comprehensive protocol for ventilator weaning and extubation on reintubation rate and hospital mortality

Reintubation is associated with a fivefold increase in death [10]. Low hospital mortality in our study seems to mainly result from low reintubation rates. Among patients with PERF, reintubated patients had equivalent outcomes compared with patients without reintubation. As a result, hospital mortality was reduced in our study, and the lower rate of reintubation and hospital mortality in our study are thought to be due to the comprehensive protocol for ventilator weaning and extubation. Each item in both weaning and extubation processes has already been reportedly used in previous studies. However, we believe that the comprehensive protocol per se, including all the processes of ventilator weaning and extubation, was effective in improving the outcomes by their synergistic effects. A systematic approach to ventilator weaning and extubation has been reported [3, 23], but this is the first report to investigate the effectiveness of a comprehensive protocol for ventilator weaning and extubation including prophylactic NPPV and evaluation after extubation.

### Implication of checklists on the comprehensive protocol for ventilator weaning and extubation

#### The evaluation of extubation to prevent PERF

The extubation process is a critical component of respiratory care in patients who receive MV under tracheal intubation. However, the extubation process has not received the same attention as the process of ventilator weaning. Physicians do not always have similar judgment skills regarding extubation. This might be one of the reasons for the wide variation of reintubation rates among different institutions (range, 5–25%) [1–3, 10, 11, 17]. Therefore, standardization of the extubation process as well as ventilator weaning is expected to minimize variability in judgment among physicians and the risk of PERF. To this end, we developed a comprehensive protocol for guiding the decision-making process regarding ventilator weaning and extubation.

Our extubation checklist included airway protection and patency factors. Cough reflex [7] and gag reflex [24] as factors of airway protection have been reported. The level of consciousness [4] and the cuff leak test [8] as factors of airway patency have been reported. Each factor has been reported to be an important predictor of extubation failure. Moreover, similar to the other reports [9, 25], the synergistic effect of checking predictors of PERF may play a role in our results.

#### The effect of prophylactic NPPV in high risk of PERF

NPPV has not been effective when used routinely after extubation in unselected patients [26]. However, Nava et al. reported that early application of NPPV was effective in preventing PERF in an at-risk population [27]. Ferrer et al. also reported that early use of NPPV averted PERF and decreased mortality among patients at increased risk of PERF [11]. Therefore, extubation processes including prophylactic NPPV may be effective for reducing PERF and reintubation. In this study, reintubation, PERF, and mortality rates of patients who had high risk of PERF and received prophylactic NPPV were not different from those of patients who received conventional oxygen therapy. These findings are consistent with those of previous reports [11, 27].

“Cardiac failure as the reason for intubation” was the most common reason for undergoing prophylactic NPPV. Some studies reported that NPPV benefited patients with cardiac failure [28, 29]. The favorable results of our study might have been partially because patients with congestive heart failure were selected as being at high risk of PERF by the protocol and underwent prophylactic NPPV.

Meanwhile, risk factors for PERF have been recently reported [7, 24, 25, 27, 30]. Hernández et al. reported that risk factors included older age, APACHE II greater than 12, body mass index greater than 30 kg/m^2^, inadequate secretions management, difficult or prolonged weaning, more than one comorbidity, heart failure as an indication for mechanical ventilation, moderate to severe chronic obstructive pulmonary disease, airway patency problems, and prolonged mechanical ventilation [30]. In our study, we investigated only three risk factors of PERF. If our third checklist had had items that could be used to detect patients who required prophylactic NPPV, some of the 23 patients with PERF in conventional O_2_ therapy group might have received prophylactic NPPV and avoided PERF.

#### The evaluation after extubation to prevent reintubation and to reduce mortality

It has been reported that the mortality rate increased in proportion to the interval between extubation and reintubation [31]. Thus, the timing of reintubation also seems to influence hospital mortality. In our study, patients were evaluated by the use of the fourth risk assessment checklist once every hour after extubation. The status of patients was rapidly assessed without delay in this protocol. Outcomes of patients with PERF were not significantly different from those of those without PERF. It also might be said that the fourth checklist is the one effect that reduced the risk considerably. It is quite possible that the evaluation after extubation risk assessment contributed to the reduction of overall mortality. Or it is possible to say that the first three checklists might have accelerated the detection of those patients who were in need of reintubation which were identified by the fourth checklist.

### Limitations

There are limitations to the present study. First, this study used an observational and non-interventional design, which entails the risk of bias. Second, this study was conducted at a single center, and the results might not be generalizable to other institutions. Third, in this study, unfortunately, blinding of the investigator could not have been done. Moreover, there is difficulty in achieving blinding of the attending physicians in this type of clinical study; this might have led to potential performance bias. Fourth, generally, a before-and-after design is used to evaluate the effectiveness of a protocol in a single center study. However, we have administered our advanced emergency and critical care center in 2007 and have simultaneously used this protocol. Thus, we could not compare before-and-after designs. Fifth, the use of high flow nasal cannula oxygenation with critically ill adults has been increasing dramatically [32] and reported to be effective in patients after planned extubation [21, 30]. Unfortunately, its use was not a standard treatment approach in adults still, when we developed this protocol. High flow nasal cannula oxygenation may be increasingly used for patients with high risk of PERF [32, 33]. A multicenter clinical trial is needed to demonstrate the benefits of a comprehensive protocol for ventilator weaning and extubation.

## Conclusion

A comprehensive protocol for ventilator weaning and extubation in critically ill patients may prevent PERF and reintubation and reduce mortality.

## Supplementary information

**Additional file 1.** Outcomes in patients with and without reintubation after PERF.

**Additional file 2.** Comparison of patient characteristics with prophylactic NPPV and conventional oxygen (O2) therapy.

**Additional file 3.** Comparison of outcome variables with prophylactic NPPV and conventional oxygen (O2) therapy.

## Data Availability

The dataset supporting the conclusions of this article is included within the article.

## Abbreviations

ABG: Arterial blood gas
APACHE II: Acute Physiology and Chronic Health Evaluation II
ARDS: Acute respiratory distress syndrome
CCC: Critical care center
COPD: Chronic obstructive pulmonary disease
FiO_2_: Fraction of inspired oxygen
GCS_*T*_: Glasgow Coma Scale under tracheal intubation
HR: Heart rate
MAP: Mean arterial pressure
MV: Mechanical ventilation
NPPV: Non-invasive positive pressure ventilation
PaCO2: Partial pressure of carbon dioxide
PaO_2_: Partial pressure of arterial oxygen
PEEP: Positive end-expiratory pressure
PERF: Post-extubation respiratory failure
RR: Respiratory rate
RSBI: Rapid shallow breathing index
SBT: Spontaneous breathing trial
SOFA_*T*_ score: Sequential organ failure assessment score under tracheal intubation
SpO_2_: Arterial oxygen saturation
