# Supplementary material for: A comprehensive protocol for ventilator weaning and extubation: a prospective observational study
Source: J Intensive Care. 2019 Nov 6;7:50. doi: 10.1186/s40560-019-0402-4 (PMC6833251; doi:10.1186/s40560-019-0402-4)
Supplement: Supplementary file 1 — Additional file 1. Outcomes in patients with and without reintubation after PERF. [file 40560_2019_402_MOESM1_ESM.docx]

**Additional file 1** Outcomes in patients with and without reintubation after PERF.

|  | PERF | Patients with reintubation | | | Patients without reintubation | P-values |
| --- | --- | --- | --- | --- | --- | --- |
|  | (n = 24) | (n = 13) | | | (n = 11) |  |
| Reasons for PERF, n (%) |  | |  | |  | 0.9 |
| Upper airway obstruction | 3 | | 1 | 2 | |  |
| Incapacity to remove secretions | 12 | | 7 | 5 | |  |
| Refractory hypoxia | 6 | | 3 | 3 | |  |
| Atelectasis | 1 | | 1 | 0 | |  |
| Exacerbation of heart failure | 2 | | 1 | 1 | |  |
| CCC stay (days) | 25 (16.8–47) | | 47 (25-52) | 17 (12-23.5) | | 0.003 |
| Hospital stay (days) | 44 (25.8–57.8) | | 52 (41-93) | 31 (19.5-41) | | 0.07 |
| Tracheotomy, n (%) | 10 (42) | | 10 (77) | 0 (0) | | 0.0001 |
| 28-d mortality | 1 (4.1) | | 1 | 0 | | 1 |
| 60-d mortality | 1 (4.1) | | 1 | 0 | | 1 |
| Hospital mortality | 4 (16.7) | | 3 | 1 | | 0.5 |

Data are presented as median and interquartile range or number (percentage). *PERF* post-extubation respiratory failure, *CCC* critical care center, *28-d mortality* 28-day mortality after admission, *60-d mortality* 60-day mortality after admission
