# Supplementary material for: A comprehensive protocol for ventilator weaning and extubation: a prospective observational study
Source: J Intensive Care. 2019 Nov 6;7:50. doi: 10.1186/s40560-019-0402-4 (PMC6833251; doi:10.1186/s40560-019-0402-4)
Supplement: Supplementary file 2 — Additional file 2. Comparison of patient characteristics with prophylactic NPPV and conventional oxygen (O2) therapy. [file 40560_2019_402_MOESM2_ESM.docx]

**Additional file 2** Comparison of patient characteristics with prophylactic NPPV and conventional oxygen (O2) therapy

|  | Prophylactic NPPV | Conventional O2 therapy | | | P-values |
| --- | --- | --- | --- | --- | --- |
|  | (n = 35) | | (n = 213) | |  |
| Age (years) | 67 ± 14 | 65 ± 17 | | 0.4 | |
| Women, n (%) | 7 (20) | 70 (33) | | 0.2 | |
| APACHE II score just before extubation | 14 (11–16) | 13 (10–16) | | 0.3 | |
| SOFA_T_ score just before extubation | 6 (5–7) | 5 (3–6) | | 0.009 | |
| RSBI (breaths/min/L) | 54 ± 20 | 43 ± 20 | | 0.003 | |
| Duration of mechanical ventilation (days) | 8 (4.5–12.5) | 6.5 (5–11) | | 0.3 | |
| Reason for mechanical ventilation, n (%) |  |  | | < 0.001 | |
| ARDS | 13 (37) | 146 (69) | | 0.001 | |
| Congestive heart failure | 19 (54) | 38 (18) | | < 0.001 | |
| Post-cardiac arrest syndrome | 2 (6) | 28 (13) | | 0.3 | |
| COPD exacerbation | 1 (3) | 1 (1) | | 0.3 | |
| Comorbid diseases, n (%) |  |  | | 0.3 | |
| COPD | 5 (14) | 12 (6) | |  | |
| Coronary artery disease | 8 (23) | 27 (13) | |  | |
| Chronic heart failure | 6 (17) | 19 (9) | |  | |
| Chronic renal failure | 3 (9) | 11 (5) | |  | |
| Diabetes mellitus | 10 (29) | 34 (16) | |  | |
| Hypertension | 10 (29) | 75 (35) | |  | |
| Vital signs just before extubation |  |  | |  | |
| Heart rate (beats/min) | 81 ± 16 | 89 ± 18 | | 0.02 | |
| MAP (mmHg) | 86 ± 15 | 91 ± 15 | | 0.06 | |
| RR (breaths/min) | 22 ± 7 | 19 ± 6 | | 0.02 | |
| ABG values just before extubation |  |  | |  | |
| pH | 7.46 ± 0.04 | 7.45 ± 0.04 | | 0.2 | |
| PaCO_2_ (mmHg) | 40 ± 7 | 38 ± 5 | | 0.02 | |
| PaO_2_ /FiO_2_ | 242 ± 62 | 310 ± 83 | | < 0.001 | |

Data are presented as mean ± standard deviation, median and interquartile range, or number (percentage). *APACHE Ⅱ* Acute Physiology and Chronic Health Evaluation Ⅱ, *SOFA_T_ score* sequential organ failure assessment score under tracheal intubation, *RSBI* rapid shallow breathing index, *ARDS* acute respiratory distress syndrome, *COPD* chronic obstructive pulmonary disease, *MAP* mean arterial pressure, *RR* respiratory rate, *ABG* arterial blood gas, *PaCO2* partial pressure of carbon dioxide, *PaO_2_* partial pressure of arterial oxygen, *FiO_2_* fraction of inspired oxygen
