# Supplementary material for: A comprehensive protocol for ventilator weaning and extubation: a prospective observational study
Source: J Intensive Care. 2019 Nov 6;7:50. doi: 10.1186/s40560-019-0402-4 (PMC6833251; doi:10.1186/s40560-019-0402-4)
Supplement: Supplementary file 3 — Additional file 3. Comparison of outcome variables with prophylactic NPPV and conventional oxygen (O2) therapy. [file 40560_2019_402_MOESM3_ESM.docx]

**Additional file 3** Comparison of outcome variables with prophylactic NPPV and conventional oxygen (O2) therapy

|  | Prophylactic NPPV | Conventional O2 therapy | P-values |
| --- | --- | --- | --- |
|  | (n = 35) | (n = 213) |  |
| PERF, n (%) | 1 (2.8) | 23 (10.8) | 0.2 |
| Reintubation, n (%) | 1 (2.8) | 12(5.6) | 1.0 |
| CCC stay (days) | 21 (12–42) | 20 (12.5–32) | 0.6 |
| Hospital stay (days) | 45 (27.5–57.5) | 33 (20–51.8) | 0.09 |
| 28-d mortality | 0 (0) | 3 (1.4) | 1.0 |
| 60-d mortality | 1 (2.8) | 10 (4.7) | 1.0 |
| Hospital mortality | 3 (8.6) | 14 (6.6) | 0.7 |

Data are presented as median and interquartile range or number (percentage). *PERF* post-extubation respiratory failure, *CCC* critical care center, *28-d mortality* 28-day mortality after admission, *60-d mortality* 60-day mortality after admission
